# Integrative epigenome and transcriptome analyses reveal transcriptional programs differentially regulated by ASCL1 and NEUROD1 in small cell lung cancer

**DOI:** 10.1038/s41388-025-03481-2

**Published:** 2025-07-01

**Authors:** Hiroshi Takumida, Akira Saito, Yugo Okabe, Yasuhiro Terasaki, Yu Mikami, Hidenori Tanaka, Masami Suzuki, Yu Hamaguchi, Chao Zeng, Michiaki Hamada, Hiroshi I. Suzuki, Hidenori Kage, Masafumi Horie

**Affiliations:** 1 Department of Respiratory Medicine, Graduate School of Medicine, The University of Tokyo, Tokyo, 113-0033 Japan; 2 Department of Analytic Human Pathology, Nippon Medical School, Tokyo, 113-8603 Japan; 3 Division of Pathology, Nippon Medical School Hospital, Tokyo, 113-8603 Japan; 4 Department of Otorhinolaryngology-Head and Neck Surgery, Graduate School of Medicine, The University of Osaka, Osaka, 565-0871 Japan; 5 Faculty of Science and Engineering, Waseda University, Tokyo, 169-8555 Japan; 6 AIST-Waseda University Computational Bio Big-Data Open Innovation Laboratory (CBBD-OIL), National Institute of Advanced Industrial Science and Technology, Tokyo, 169-8555 Japan; 7 Division of Molecular Oncology, Center for Neurological Diseases and Cancer, Nagoya University Graduate School of Medicine, Nagoya, 466-8550 Japan; 8 Institute for Glyco-core Research (iGCORE), Nagoya University, Nagoya, 464-8601 Japan; 9 Center for One Medicine Innovative Translational Research (COMIT), Nagoya University, Nagoya, 464-8601 Japan; 10 Inamori Research Institute for Science (InaRIS), Kyoto, 600-8411 Japan; 11 Department of Molecular and Cellular Pathology, Graduate School of Medical Sciences, Kanazawa University, Kanazawa, 920-0934 Japan; 12 Division of Molecular and Genomic Pathology, Department of Pathology, Kobe University Graduate School of Medicine, Kobe, 650-0017 Japan

**Keywords:** Cancer genetics, Small-cell lung cancer, Oncogenes, Tumour heterogeneity

## Abstract

Small cell lung cancer (SCLC), an aggressive neuroendocrine carcinoma, has an extremely poor prognosis. ASCL1 and NEUROD1 are key regulators of neuroendocrine features, and previous studies have suggested that SCLC plasticity occurs during the transition from ASCL1-positive (SCLC-A) to NEUROD1-positive (SCLC-N) subtypes. In this study, we attempted to understand the transcriptional programs governed by ASCL1 and NEUROD1 to identify markers of SCLC plasticity. Immunohistochemistry and epigenome and transcriptome analyses in ASCL1/NEUROD1 double-positive SCLC cells (SCLC-A/N) revealed co-expression of ASCL1 and NEUROD1 in almost half of SCLC cases. Genome-wide profiling of histone modifications, ASCL1 and NEUROD1 binding sites, and gene co-expression patterns revealed that both ASCL1 and NEUROD1 are active in SCLC-A/N and regulate partially distinct target genes. Furthermore, SCLC-A/N exhibited characteristics that were intermediate between SCLC-A and SCLC-N subtypes. *NEUROD1* knockout, followed by RNA-seq, suggested an association between NEUROD and NHLH transcription factors that might shape the NEUROD1-mediated regulatory network. Small RNA-seq further indicated that miR-139-5p is specifically expressed in NEUROD1-positive SCLC, and transcriptomic studies suggested that miR-139-5p might regulate an array of pathologically relevant genes in collaboration with other NEUROD1-associated miRNAs. Our integrative analyses provide deeper insights into SCLC heterogeneity and multi-layered transcriptional programs differentially governed by ASCL1 and NEUROD1.

## Introduction

Small cell lung cancer (SCLC) is an aggressive neuroendocrine tumour that accounts for approximately 15% of lung cancer cases [1, 2]. Subtyping has emerged as a key concept in advancing personalised medicine for SCLC, Currently, SCLC is classified into four subtypes based on the expression of master transcription factors: Achaete-scute complex homologue 1 (ASCL1)-positive (SCLC-A), Neuronal differentiation 1 (NEUROD1)-positive (SCLC-N), POU class 2 homeobox 3 (POU2F3)-positive (SCLC-P), and Yes-associated protein 1 (YAP1)-positive (SCLC-Y) [3, 4]. Early morphological analyses of cell lines initially led to the identification of two major subtypes [5], a distinction later supported by the observation that ASCL1 and NEUROD1 were highly expressed in these respective subtypes [6]. Subsequently, a third group was identified, characterised by low expression of both ASCL1 and NEUROD1, but with elevated expression of either YAP1 [4] or POU2F3 [7]. These subtypes lack neuroendocrine features and represent non-neuroendocrine variants of SCLC.

The master transcription factor ASCL1 is indispensable for pulmonary neuroendocrine cell differentiation [8, 9], and targeted disruption of *Ascl1* has been shown to abrogate tumour formation in a mouse model of SCLC [10]. SCLC-A represents a prototypic subtype and constitutes most SCLC cases, although ASCL1-negative SCLC subtypes have been described [3, 11].

Neuronal differentiation 1 (NEUROD1) is another essential transcription factor related to neuroendocrine features. Studies using genetically engineered mouse models of SCLC have suggested that the SCLC-N subtype of SCLC arises from SCLC-A, which is driven by the MYC proto-oncogene, a bHLH transcription factor (MYC) [12, 13]. Single-cell transcriptomics studies of human SCLC have suggested the cellular transition of SCLC-A to SCLC-N [14, 15]. Moreover, immunohistochemical studies have demonstrated that ASCL1 and NEUROD1 could occasionally be co-expressed in 30 to 40% of human SCLC tissues, implying cellular plasticity between SCLC-A and SCLC-N [16–18].

Previous studies, including ours, have demonstrated that ASCL1 collaborates with several key transcription factors such as NK2 homeobox 1 (NKX2-1), prospero homeobox 1 (PROX1), and E74 like ETS transcription factor 3 (ELF3) [10, 11, 19–22], and participates in transcriptional regulation associated with super-enhancers (SEs). In contrast, NEUROD1-mediated transcriptional control and its association with other transcription factors have not been fully characterised [10, 23, 24].

MicroRNAs (miRNAs), which are expressed in a cancer type-specific manner, are involved in post-transcriptional gene regulations by binding to the 3’ untranslated regions of the target mRNAs [25]. Recently, we reported that ASCL1 regulates a subset of miRNAs (for example, miR-375 and miR-7), thereby contributing to shaping the molecular heterogeneity of SCLC [21]. Meanwhile, NEUROD1-mediated regulation of miRNAs has not yet been elucidated.

In the current study, given that SCLC-A could convert into SCLC-N through a continuous process as suggested by cellular and murine models [12–15], we assumed that ASCL1/NEUROD1 double-positive SCLC corresponds to an intermediate state, which could provide a clue to understand SCLC plasticity. Additionally, we reasoned that such ASCL1/NEUROD1 double-positive cells might be suitable for analysing differential transcriptional regulations and their potential interactions under the same culture conditions. We performed cleaved under targets and tagmentation (CUT&Tag) assay, RNA-sequencing (RNA-seq), and small RNA-seq to identify epigenome and transcriptome features regulated by ASCL1 and NEUROD1.

## Materials and methods

### Public datasets

The public datasets analysed in this study are shown in Table S1. The SCLC cell lines of Cance Cell Line Encyclopaedia (CCLE) [26] analysed in this study are listed in Table S2.

### Cell cultures

Human SCLC cell lines DMS53, DMS454, and H2066 were purchased from ATCC (Manassas, VA, USA). Additionally, Lu134A and WA-hT cells were obtained from RIKEN BRC (Tsukuba, Japan), and CORL279 was purchased from ECACC (Porton Down, Salisbury, UK). H2066 cells were cultured in DMEM/F12 medium supplemented with 5% foetal bovine serum (FBS) and HITES, which includes 0.005 mg/ml insulin, 0.01 mg/ml transferrin, 30 nM sodium selenite, 10 nM hydrocortisone, 10 nM β-estradiol, and an additional 2 mM L-glutamine (final L-glutamine concentration: 4.5 mM). CORL279 cells were cultured in RPMI medium supplemented with 10% FBS. The remaining cell lines were cultured as previously described [20].

### Gene editing

CRISPR/Cas9-mediated gene editing was performed as follows. Ribonucleoprotein complexes were prepared by mixing 100 pmol of single guide RNA (sgRNA) targeting the sequences listed in (Table S3) and 40 pmol of SpCas9 2NLS Nuclease (Synthego, Redwood City, CA, USA) and were delivered to the suspension of 3 × 10^6^ cells by electroporation using the Invitrogen Neon Transfection System (Thermo Fisher Scientific, Waltham, MA, USA) under the following conditions: 1600 V, 20 ms, 1 pulse. Gene editing by CRISPR/Cas9 was confirmed using Sanger sequencing of the relevant sites.

### Transfection of miRNA mimics

Hsa-miR-139-5p and negative control miRCURY LNA miRNA mimics were purchased from QIAGEN (Hilden, Germany) and were transfected by electroporation under the same conditions as those used for gene editing.

### CUT&Tag

Library preparation and data processing for CUT&Tag were performed as described previously [20, 27, 28]. The pooled libraries were prepared using Lu134A, DMS53, DMS454, and WA-hT cells (1 × 10^5^) and were sequenced on Novaseq X Plus (Illumina, San Diego, CA, USA) using 150 bp paired-end reads. Alignment, peak calling, and the definition of SEs and typical enhancers (TEs) were performed as previously described [20, 21]. The antibodies used for CUT&Tag are shown in Table S4. Mapped sequence data were visualised using Integrative Genomics Viewer [29]. Raw sequencing data were deposited in the DNA Data Bank of Japan (DRA012871) and the Gene Expression Omnibus repository (GSE277353).

### RNA-seq and small RNA-seq analyses

RNA and miRNA were isolated using the miRNeasy kit (QIAGEN). RNA-seq and small RNA-seq were performed on the Illumina NovaSeq 6000 system (Illumina). For RNA-seq, library preparation was performed using the NEBNext Poly(A) mRNA Magnetic Isolation Module and the NEBNext Ultra II Directional RNA Library Prep Kit (New England Biolabs, Ipswich, MA, USA), and 150 bp paired-end reads were obtained, yielding 6 G bases and 40 million reads (20 million read pairs) per sample. For small RNA-seq, libraries were prepared using the NEBNext Multiplex Small RNA Library Prep Kit for Illumina (New England Biolabs), and 50 bp single-end reads were obtained, yielding 0.5 G bases and 10 million reads per sample. Sequencing data were analysed using CLC Genomics workbench software (QIAGEN) and deposited in GSE270263. Predicted target genes with conserved sites for each miRNA were obtained from TargetScan (release 8.0) [30].

### Immunoblot analysis

Immunoblotting was performed as described previously [21]. The antibodies are listed in Table S4.

### Immunohistochemistry

Lung samples were obtained from 15 patients who were pathologically diagnosed with SCLC, each with a tumour cell percentage ≥50%. The study protocol for obtaining the human lung specimens was approved (M-2021-020) by the Ethics Review Committee of Nippon Medical School. Immunohistochemistry was performed as described previously [21]. The antibodies are shown in Table S4. Immunostaining was considered positive if more than 50% of the tumour cells were stained, partially positive if 10% to 50% were stained, and negative if less than 10% were stained.

### Statistical analysis

Principal component analysis was performed using R (version 4.2.2). Pearson’s correlation coefficient (r) was calculated for correlation analyses. The Wilcoxon rank test was used to compare different gene sets.

## Results

### ASCL1 and NEUROD1 are co-expressed in a subset of SCLC

**Fig. 1:**
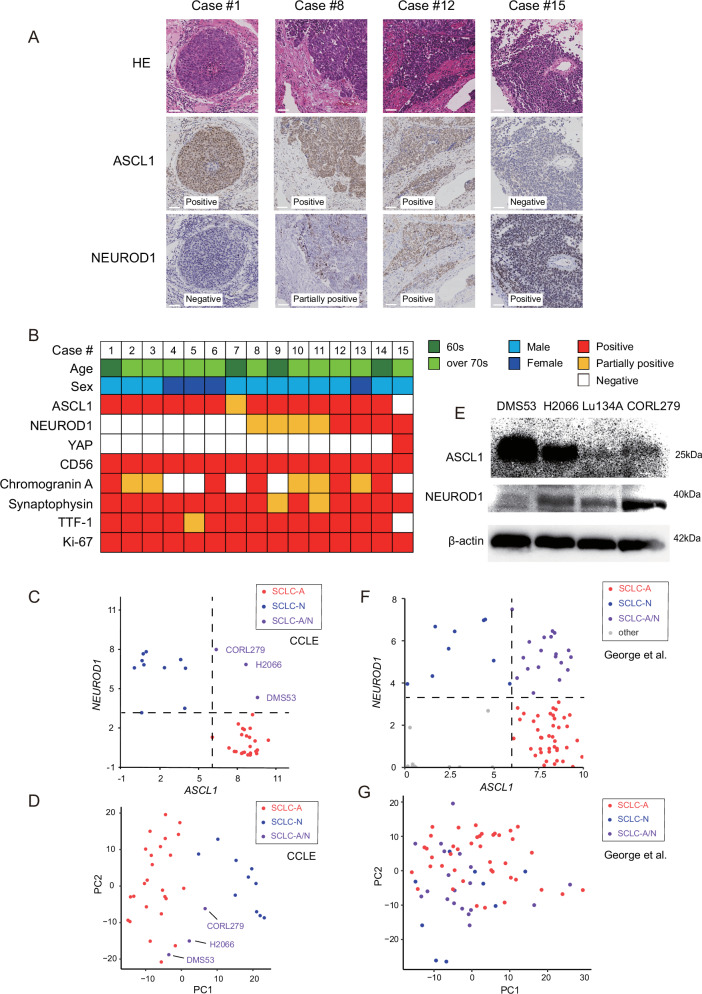
ASCL1 and NEUROD1 are co-expressed in a subset of SCLC. **A** Representative pathological images showing four hematoxylin and eosin staining patterns, as well as immunohistochemistry for ASCL1 and NEUROD1. Scale bar: 50 μm. **B** Clinical characteristics of the 15 SCLC cases and immunostaining patterns of the indicated genes. **C** Scatter plot showing *ASCL1* (x-axis) and *NEUROD1* (y-axis) expression in 38 SCLC cell lines. Expression levels are shown as log_2_ (1 + RPKM [reads per kilobase of transcript per million reads mapped]). Dashed lines indicate the minimum expression levels of ASCL1 and NEUROD1 used in the current SCLC classification (SCLC-A or SCLC-N). Red, blue, and purple marks represent SCLC-A, SCLC-N, and SCLC-A/N, respectively. **D** Principal component analysis based on the expression of 1628 transcription factors. Red, blue, and purple marks represent SCLC-A, SCLC-N, and SCLC-A/N, respectively. Scores of components 1 (*x*-axis: PC1) and 2 (*y*-axis: PC2) are shown. The distance between samples indicates the similarity of gene expression profiles. **E** Immunoblotting for ASCL1 and NEUROD1 in SCLC-A/N subtype cell lines. Beta-actin was used as the loading control. **F** Scatter plot showing *ASCL1* (x-axis) and *NEUROD1* (y-axis) expression in 81 SCLC tissue samples. Expression levels are shown as log_2_ (1 + FPKM [fragments per kilobase of exon per million mapped reads]). Dashed lines indicate the minimum expression levels of ASCL1 and NEUROD1 used in the current SCLC classification (SCLC-A or SCLC-N). Red, blue, purple and grey marks represent SCLC-A, SCLC-N, SCLC-A/N and other respectively. **G** Principal component analysis based on the expression of 1,628 transcription factors. Red, blue, and purple marks represent SCLC-A, SCLC-N, and SCLC-A/N, respectively. Scores of components 1 (*x*-axis: PC1) and 2 (*y*-axis: PC2) are shown. The distance between samples indicates the similarity of gene expression profiles. Compared to cell lines **D**, the separation between subtypes in clinical samples was less distinct.

First, we performed immunostaining for ASCL1 and NEUROD1 on SCLC tissue samples, collected from the primary tumour site, of 15 patients between 2019 and 2022 (Table S5), and observed that 14 samples were positive for ASCL1 (Fig. 1A, B). One exceptional case was negative for ASCL1 but was positive for NEUROD1 (Case #15, Fig. 1A). In line with previous studies [16–18], 7 of the 14 ASCL1-positive cases were positively stained for both ASCL1 and NEUROD1 to varying degrees (Fig. 1B), and double-positive cells were distributed in part or the whole of the tumour area (Fig. 1A). At least 2 out of 3 neuroendocrine markers (CD56, chromogranin A, and synaptophysin) were positive in all cases **(**Fig. 1B**)**.

Next, we examined the expression patterns of *ASCL1* and *NEUROD1* in different SCLC cell lines (Fig. 1C). We exploited the CCLE database [26] and compared the gene expression profiling data of 38 SCLC cell lines, previously classified as either SCLC-A or SCLC-N [11], all of which expressed *ASCL1*, *NEUROD1*, or both (Table S2). In concordance with histological observations on tissue samples, a subset of SCLC cell lines positive for NEUROD1 (CORL279, H2066, and DMS53; 3 of 38 (8%)), which had been previously assigned as SCLC-N [11], showed *ASCL1* expression comparable to that in SCLC-A (Fig. 1C). We designated the ASCL1/NEUROD1 double-positive SCLC subtype SCLC-A/N and aimed to clarify the transcriptional regulation by *ASCL1* and/or *NEUROD1* in this cell type. We previously suggested that the heterogeneity of SCLC cell lines may be attributable to differences in the expression patterns of key transcription factors [4]. To illustrate the transcriptomic features, we performed a principal component analysis based on the expression of 1628 human transcription factors [31] (Table S6). The SCLC-A and SCLC-N cell lines were separated, indicating distinct regulation mechanisms by ASCL1 or NEUROD1 (Fig. 1D). We further confirmed that all four SCLC‑A/N cell lines, including Lu134A, which was used in subsequent experiments, express both ASCL1 and NEUROD1 proteins, although expression levels varied across the lines **(**Fig. 1E**)**. This observation highlights the heterogeneity of ASCL1/NEUROD1 co‑expression in SCLC, a feature we further explore in subsequent analyses.

To determine whether these transcriptional relationships extend to clinical samples, we analysed a dataset from George et al., which included transcriptomic data from 81 tissue samples obtained from patients with SCLC [32]. Using the same classification criteria applied to cell lines, we identified SCLC-A (41 of 81, 51%), SCLC-N (9 of 81, 11%), SCLC-A/N (19 of 81, 23%), and other subgroups (12 of 81, 15%) **(**Fig. 1F**)**. Principal component analysis based on transcription factor expression revealed that SCLC-A/N tumours was positioned between SCLC-A and SCLC-N, mirroring the pattern observed in cell lines, although the separation between subtypes was less distinct in clinical samples **(**Fig. 1G**)**.

### Chromatin modifications and transcriptional regulations by ASCL1 and NEUROD1 in SCLC cell lines

**Fig. 2:**
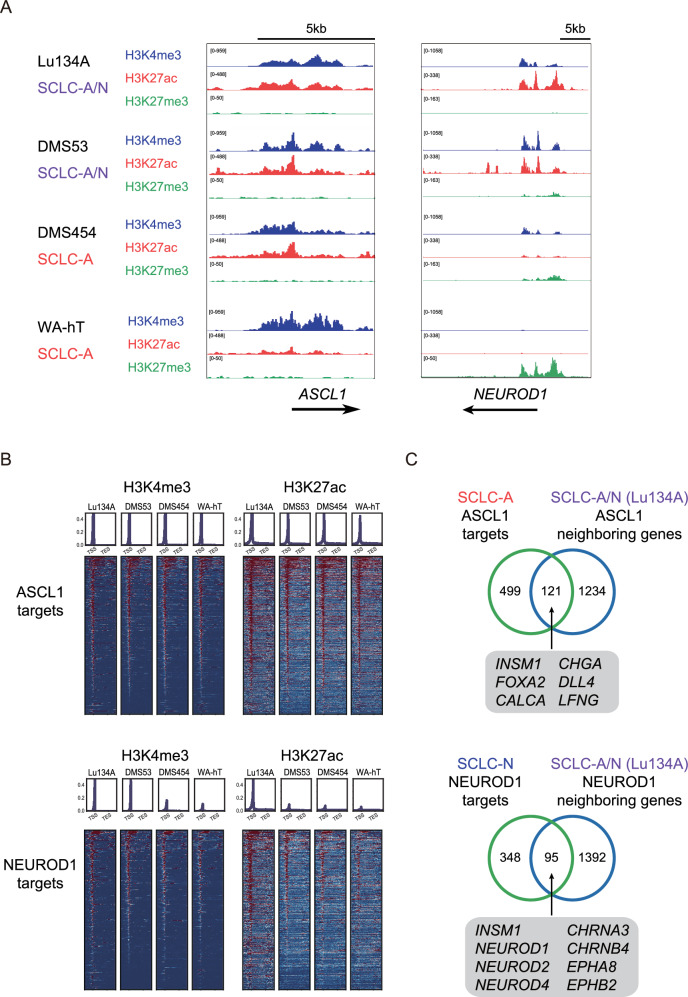
Chromatin modifications and transcriptional regulations by ASCL1 and NEUROD1 in SCLC cell lines. **A** H3K4me3, H3K27ac, and H3K27me3 signals (expressed on the vertical axis) in the genomic regions around the *ASCL1* and *NEUROD1* gene loci of Lu134A, DMS53, DMS454, and WA-hT cells. The arrows indicate the transcripts of *ASCL1* and *NEUROD1*. Note that the starting point of each arrow corresponds to the transcription start site (TSS). **B** Distributions of H3K4me3 and H3K27ac signals within the intervals of 3.0 kb upstream of TSSs, gene bodies, and 3.0 kb downstream of transcription end sites (TESs) of the ASCL1 or NEUROD1 target genes in DMS53, DMS454, Lu134A, and WA-hT. The values on the *Y*-axis of the upper panels indicate normalised read counts. **C** Venn diagram showing overlap of known target genes of ASCL1 and NEUROD1 identified in SCLC-A and SCLC-N cell lines, respectively, and neighbouring genes of CUT&Tag peaks identified using CUT&Tag assay for ASCL1 and NEUROD1 in Lu134A cells. Numbers of genes are indicated.

We used SCLC-A/N (Lu134A and DMS53) and SCLC-A (DMS454 and WA-hT) cell lines for further epigenome analyses. To evaluate the genome-wide histone modifications, we performed CUT&Tag assay [20, 27, 28] for H3 tri-methylation at lysine 4 (H3K4me3), H3K27ac, and H3K27me3. In line with our previous observation that all these cell lines express ASCL1 [20], broad distributions of both H3K4me3 and H3K27ac were noted around the transcription start site (TSS) of *ASCL1*, which contrasted with the absence of H3K27me3-defined repressive marks (Fig. 2A, left). Furthermore, we observed high H3K4me3 and H3K27ac signals around the TSS of *NEUROD1* in Lu134A and DMS53 (SCLC-A/N) while not in DMS454 and WA-hT (SCLC-A) (Fig. 2A, right), indicating that both *ASCL1* and *NEUROD1* are transactivated in SCLC-A/N. In agreement with these results, RNA-seq analysis showed that Lu134A cells highly expressed both *ASCL1* and *NEUROD1*, whereas only *ASCL1* was detected in WA-hT cells (Supplementary Fig. S1A).

We next investigated H3K4me3, H3K27ac, and H3K27me3 histone marks around the TSSs of the known target genes of ASCL1 and NEUROD1, which were identified in SCLC-A and SCLC-N cell lines in a previous report [10] **(**Table S7 and Fig. 2B). We observed high H3K4me3 and H3K27ac signals around the TSSs of ASCL1 targets in all four cell lines (Fig. 2B). In contrast, H3K4me3 signals for NEUROD1 targets were only noted in SCLC-A/N cell lines. Moreover, H3K27ac marking was prominent around the TSSs of NEUROD1 targets in Lu134A cells, suggesting that both ASCL1 and NEUROD1 are functionally active in Lu134A cells. Few H3K27me3 signals were observed around the TSSs of either ASCL1 or NEUROD1 target genes in all four cell lines, implying a minor role for H3K27me3-mediated gene repression (Supplementary Fig. S1B).

To further assess the direct transcriptional regulations by ASCL1 and NEUROD1 in SCLC-A/N, we conducted CUT&Tag to identify genome-wide binding profiles of ASCL1 and NEUROD1 in Lu134A cells. Motif analysis confirmed the preferential binding of ASCL1 and NEUROD1 to their cognate motifs (Supplementary Fig. S2A). The associated motifs for ASCL1 and NEUROD1 were distinct from each other. With histone modification patterns (Fig. 2B), ASCL1 and NEUROD1 seemed to be actively involved in transcriptional control in Lu134A cells. The CUT&Tag assay identified 1355 and 1487 neighbouring genes close to the binding peaks of ASCL1 and NEUROD1, respectively (Table S8). Half of them (657 genes) were common, suggesting combinatorial regulations by ASCL1 and NEUROD1 (Supplementary Fig. S2B).

Substantial portions of the genes neighbouring ASCL1 or NEUROD1 in Lu134A cells overlapped with the known target genes identified in SCLC-A or SCLC-N, respectively **(**Fig. 2C**)**. These included functionally important genes such as insulinoma-associated protein 1 (*INSM1*) [33], a key transcription factor crucial for neuroendocrine differentiation and known as a transcriptional target of both ASCL1 and NEUROD1 [10]. The distributions of ASCL1 and NEUROD1 CUT&Tag signals and their binding peaks for representative genes are shown in Supplementary Fig. S2C.

### NEUROD1 regulates SE-associated genes and constitutes a unique transcriptional network

**Fig. 3:**
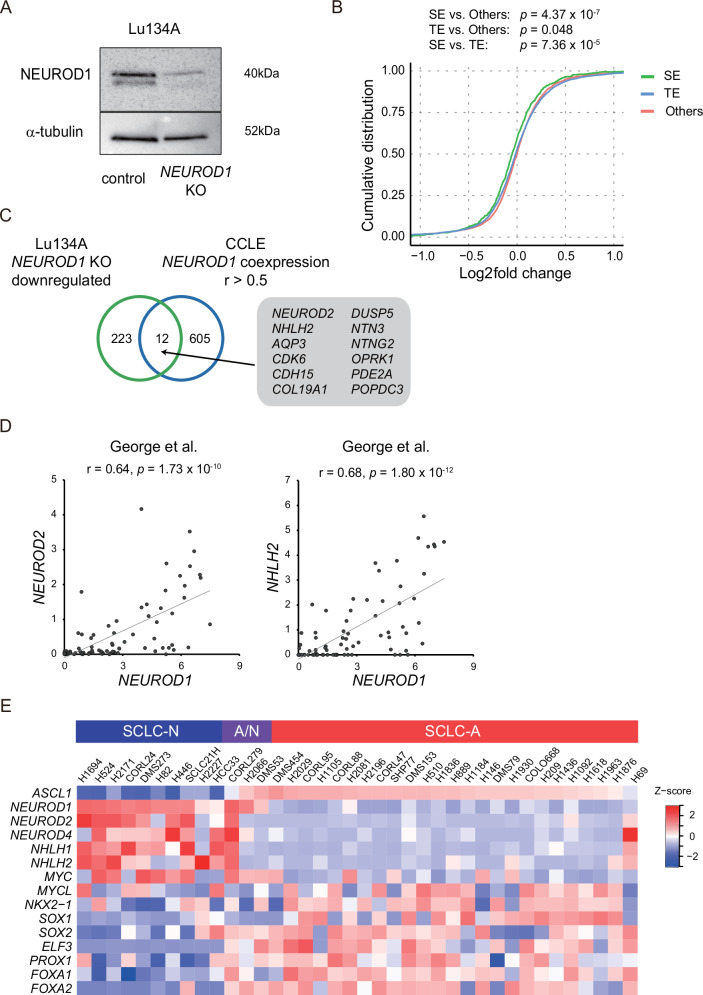
NEUROD1 regulates SE-associated genes and constitutes a unique transcriptional network. **A** Immunoblotting for NEUROD1 in Lu134 cells treated with negative control or *NEUROD1* sgRNA (*NEUROD1* knockout [KO]). Alpha-tubulin was used as the loading control. **B** Cumulative distribution plot of log_2_-transformed gene expression fold changes of SE-associated genes, TE-associated genes, and others following *NEUROD1* knockout. The genes with TPM (transcripts per million) >1 in the negative control group were analyzed. The *p*-value was calculated using the Wilcoxon rank test. SE-genes vs. others. *p* = 4.37 × 10^−7^; TE-genes vs. others, *p* = 0.048; SE-genes vs. TE-genes, *p* = 7.36 × 10^−5^. **C** Venn diagram showing overlap of downregulated genes by *NEUROD1* knockout and genes positively correlated with *NEUROD1* in 38 SCLC cell lines (*r* > 0.5). Numbers of genes are indicated. **D** Correlation of *NEUROD1* with *NEUROD2* (left) or *NHLH2* (right) in 81 SCLC tissue samples. Expression was evaluated and plotted in log_2_ (1 + FPKM) values. **E** Heatmap of the relative expression levels of key transcription factors related to SCLC-A, SCLC-A/N, and SCLC-N in 38 SCLC cell lines.

In our previous report [21], *ASCL1* knockdown followed by RNA-seq showed 409 downregulated genes in Lu134A cells (Table S9). These ASCL1-regulated genes included a substantial number of known ASCL1 target genes, including delta-like canonical Notch ligand 3 (*DLL3*), *DLL4*, and *INSM1*, whereas the effect of *ASCL1* knockdown on the *NEUROD1* target genes was not significant. We then aimed to explore NEUROD1-mediated regulations in SCLC-A/N and generated *NEUROD1*-knockout Lu134A cells using the CRISPR/Cas9 system. Efficient NEUROD1 suppression was confirmed by immunoblotting (Fig. 3A), and we conducted RNA-seq analysis in *NEUROD1*-knockout Lu134A cells. By evaluating the gene expression changes relative to untreated and negative control samples, we identified 226 upregulated and 235 downregulated genes, using the threshold of |log_2_ fold change | > 1 and excluding transcripts without available false discovery rate (FDR) q-values (Table S10).

Super-enhancers (SEs) are large genomic regions marked by high H3K27ac. We defined SE- and TE-associated genes (SE-genes and TE-genes) based on the H3K27ac CUT&Tag signals in Lu134A cells (Table S11). *NEUROD1* knockout resulted in preferential suppression of SE-associated genes compared to TE-associated genes or others (SE-genes vs. others, *p* = 4.37 × 10^−7^; TE-genes vs. others, *p* = 0.048; SE-genes vs. TE-genes, *p* = 7.36 × 10^−5^) (Fig. 3B). These results were similar to our previous findings that ASCL1-regulated genes were associated with SEs [21] and suggested that both ASCL1 and NEUROD1 participate in SE-mediated transcriptional regulation in SCLC-A/N.

Downregulated genes by *NEUROD1* knockout included the known NEUROD1 target genes, for example, *NEUROD2*, cholinergic receptor nicotinic alpha 3 subunit (*CHRNA3*), *CHRNB4*, and netrin G2 (*NTNG2*). In contrast, the effect of *NEUROD1* knockout on ASCL1 target genes was not significant. Thus, we reasoned that NEUROD1 might act independent of ASCL1 and expanded the scope of our analyses to define NEUROD1-regulated genes in SCLC-A/N and SCLC-N.

Genes positively correlated with *NEUROD1* (*r* > 0.5, 617 genes) in 38 SCLC cell lines were compared with the downregulated genes by *NEUROD1* knockout. We identified 12 common genes including *NEUROD2*, nescient helix-loop-helix 2 *(NHLH2)*, *NTNG2*, and phosphodiesterase 2 A (*PDE2A*) (Fig. 3C). Of clinical relevance, the log_2_-transformed expression of *NEUROD1* showed strong correlations with those of *NEUROD2* and *NHLH2* among 81 tissue samples of SCLC patients [32], suggesting their close relationship (Fig. 3D).

Next, we compared the expression patterns of key transcription factors in 38 SCLC cell lines. Our previous studies, along with others, demonstrated that ASCL1 is functionally associated with MYCL proto-oncogene, bHLH transcription factor (*MYCL*), *NKX2-1*, SRY-box transcription factor 1 (*SOX1*), *SOX2*, *ELF3*, *PROX1*, forkhead box A1 (*FOXA1*), and *FOXA2*, which constitute transcriptional networks via functional interactions [10, 19–22]. Expression levels of *SOX2*, *ELF3*, *PROX1*, *FOXA1*, and *FOXA2* in SCLC-A/N (CORL279, H2066, and DMS53) were relatively higher than SCLC-N and comparable to SCLC-A (Fig. 3E). In contrast, we observed higher levels of *NEUROD2*, *NEUROD4*, *NHLH1*, *NHLH2*, and *MYC* in SCLC-N as compared to SCLC-A (Fig. 3E). Moreover, these transcription factors were frequently associated with SEs in SCLC-N (Table S12). Together, NEUROD1 was strongly associated with the transcription factors of NEUROD and NHLH, which might shape the unique molecular features of NEUROD1-positive SCLC. In addition, although the expression patterns of key transcription factors indicate that SCLC-A/N is more ASCL1-leaning, clustering analysis **(**Supplementary Fig. S3A, S3B) revealed heterogeneity within this group: CORL279 clustered closer to the SCLC-N subtype, while the remaining SCLC-A/N aligned more closely with SCLC-A. This suggests that SCLC-A/N constitutes a heterogeneous population, characterised by variable degrees of ASCL1 and NEUROD1 influence.

### NEUROD1 regulates a distinct subset of miRNAs

**Fig. 4:**
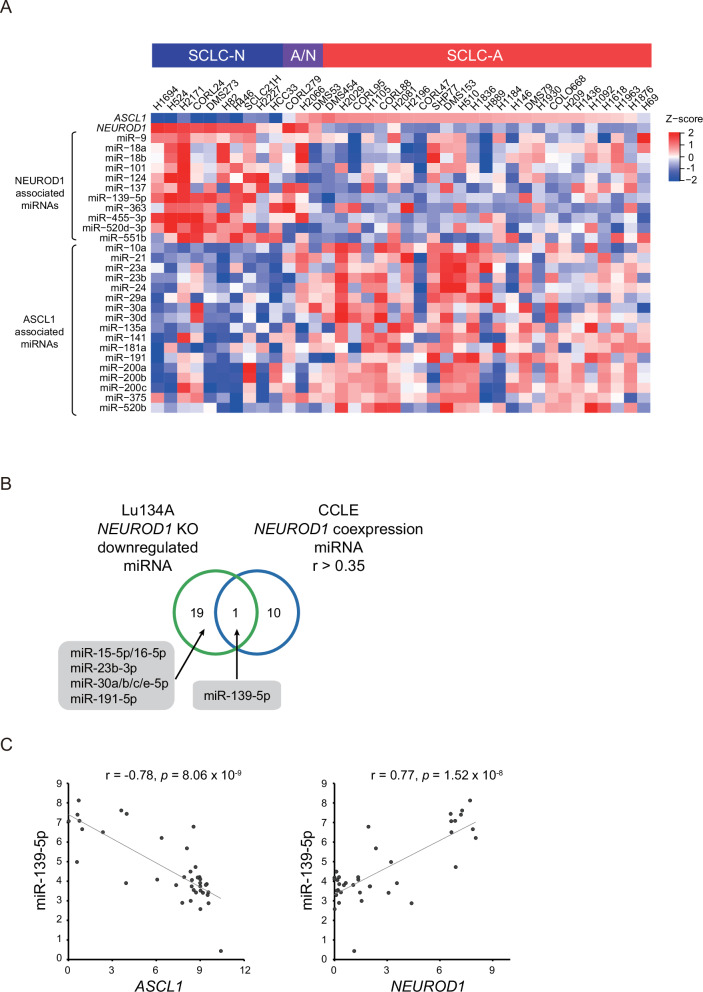
NEUROD1 regulates a distinct subset of miRNAs. **A** Heatmap of relative expression levels of *ASCL1*, *NEUROD1*, NEUROD1-associated miRNAs, and ASCL1-associated miRNAs in 38 SCLC cell lines. **B** Venn diagram showing overlap of broadly conserved miRNAs downregulated by *NEUROD1* knockout and those positively correlated with *NEUROD1* in 38 SCLC cell lines (*r* > 0.35). Numbers of miRNAs are indicated. **C** Correlations of miR-139-5p with *ASCL1* (left) and *NEUROD1* (right) in 38 SCLC cell lines. The gene expression was evaluated and plotted as log_2_ (1 + RPKM), and miRNA expression as log_2_ values of microarray signal intensity.

We next explored miRNAs associated with NEUROD1 in 38 SCLC cell lines and the broadly conserved miRNAs [30] positively correlated with *NEUROD1* (r > 0.35) included 11 miRNAs (miR-9, miR-18a, miR-18b, miR-101, miR-124, miR-137, miR-139-5p, miR-363, miR-455-3p, miR-520d-3p, and miR-551b). Similarly, we obtained a list of ASCL1-associated miRNAs, and their expression displayed contrastive expression patterns distinct from those of NEUROD1-associated miRNAs (Fig. 4A).

In addition to RNA-seq, we conducted small RNA-seq analysis following *NEUROD1* knockout in Lu134A cells. This analysis identified 20 upregulated and 47 downregulated miRNAs, using the threshold of |log_2_ fold change | > 1 and excluding data points without available FDR q-values (Table S13). We noted that 20 of 47 downregulated miRNAs were broadly conserved across most vertebrates. We then compared these 20 miRNAs with broadly conserved miRNAs positively correlated with *NEUROD1* (*r* > 0.35) in 38 SCLC cell lines (Fig. 4B). The log_2_-transformed expression levels of miR-139-5p showed a strong correlation with those of *NEUROD1* (*r* = 0.77) in contrast with the negative association with those of *ASCL1* (*r* = -0.78), suggesting that miR-139-5p could be a subtype-defining miRNA that distinguishes between SCLC-A and SCLC-N (Fig. 4C).

### PDE2A is specifically expressed in NEUROD1-positive SCLC

**Fig. 5:**
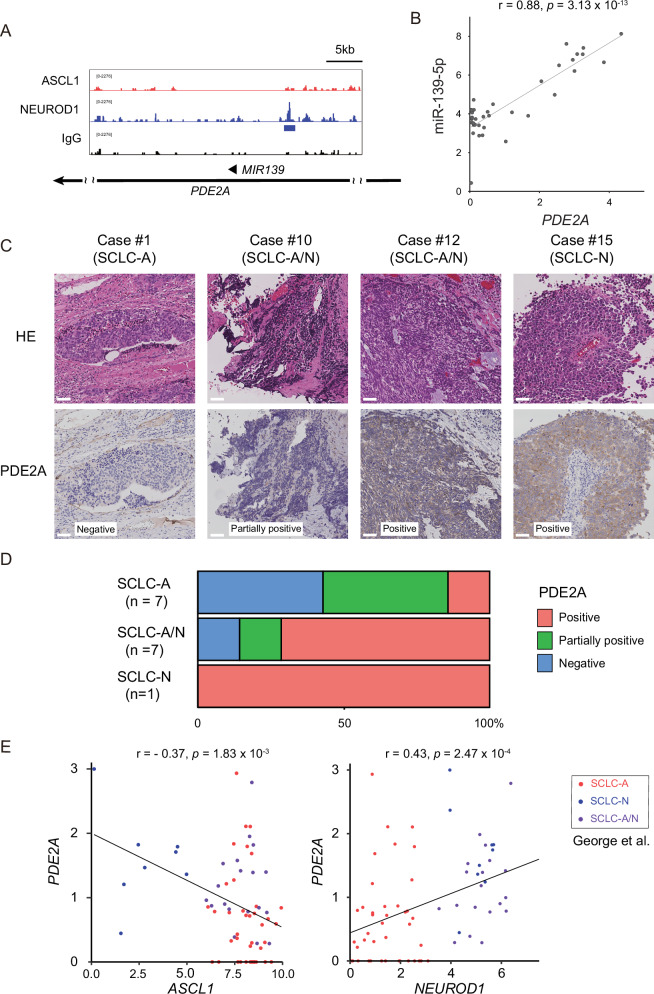
PDE2A is specifically expressed in NEUROD1-positive SCLC. **A** CUT&Tag signals for ASCL1 and NEUROD1 in the genomic regions around *PDE2A* loci in Lu134A cells. IgG signals represent the background. The blue line represents the peak. **B** Correlation between *PDE2A* and miR-139-5p in 38 SCLC cell lines. The expression levels of genes and miRNAs are shown in log_2_ (1 + RPKM) and log_2_ values of microarray signal intensity, respectively. **C** Representative pathological images showing four hematoxylin and eosin staining patterns and immunohistochemistry for PDE2A. Scale bar: 50 μm. **D** Immunohistochemistry for PDE2A and its association with ASCL1 and NEUROD1 positivity. **E** Scatter plot showing *ASCL1* and *NEUROD1* expression (*x*-axis: left for *ASCL1*, right for *NEUROD1*) and *PDE2A* expression (*y*-axis) in 81 SCLC tissue samples. Expression levels are shown as log_2_ (1 + FPKM). Red, blue, and purple points represent SCLC-A, SCLC-N, and SCLC-A/N, respectively. While the correlation coefficients indicate moderate correlations (*r* = -0.37 and 0.43), the data suggest a trend where PDE2A expression is lower in ASCL1-positive cases and higher in NEUROD1-positive cases.

CUT&Tag analysis revealed that NEUROD1 binds to the upstream region of *MIR139* in Lu134A cells, suggesting NEUROD1-mediated direct regulation (Fig. 5A). In the subsequent analyses, we focused on miR-139-5p and aimed to evaluate its expression in SCLC. First, we tested the correlation between miR-139-5p and its host gene, *PDE2A*, among 38 SCLC cell lines and observed a strong correlation between them (*r* = 0.88) (Fig. 5B), indicating that *PDE2A* could serve as a reliable surrogate marker for miR-139-5p expression. Next, we performed immunostaining for PDE2A on 15 SCLC tissue samples (Fig. 5C). Consistent with the correlation between *NEUROD1* and the miR-139-5p in the SCLC cell line (Fig. 4C), PDE2A expression was concordantly observed in NEUROD1-positive SCLC cases, and PDE2A staining was absent or showed weak positivity in NEUROD1-negative SCLC-A cases (Fig. 5D).

Consistent with our findings in SCLC cell lines, public datasets also support the observation that PDE2A is more frequently expressed in NEUROD1-positive cases and less expressed in SCLC-A cases. Among SCLC-A, SCLC-N, and SCLC-A/N, as defined in Fig. 1E, *PDE2A* expression negatively correlated with *ASCL1* and weakly positively correlated with *NEUROD1*, based on the data set of George et al. [32]. Furthermore, *PDE2A* expression tended to be lower in SCLC-A cases (Fig. 5E).

### Target gene candidates of miR-139-5p and co-targeting with other miRNAs

**Fig. 6:**
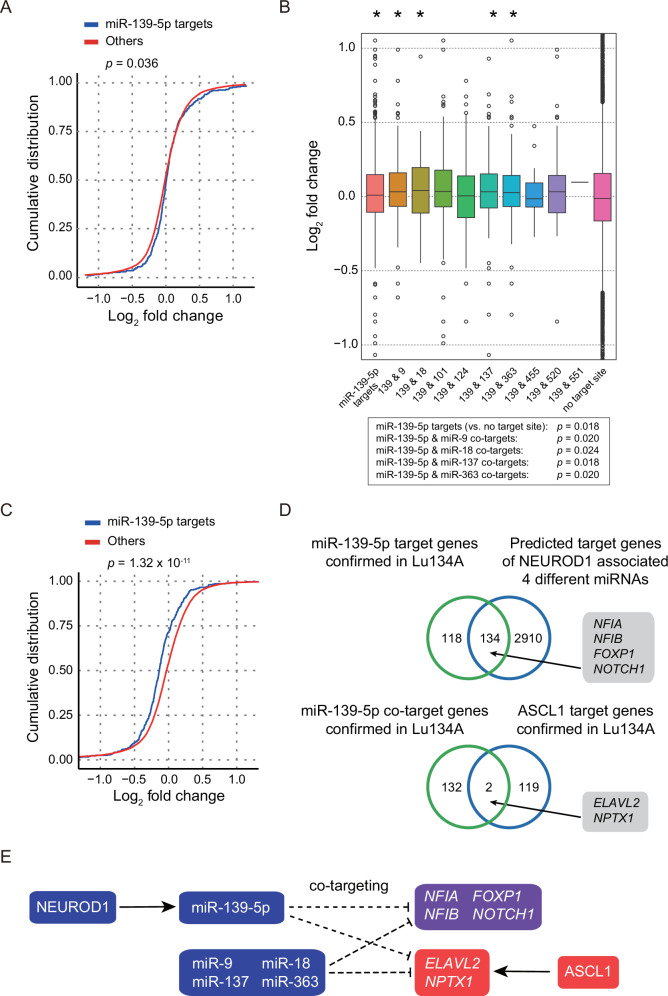
Target gene candidates of miR-139-5p and co-targeting with other miRNAs. **A** Cumulative distribution plot of log_2_-transformed gene expression fold changes of predicted miR-139-5p target genes and other genes with no target site under *NEUROD1* knockout. The genes with TPM > 1 in the negative control group were analyzed. The *p*-value was calculated using the Wilcoxon rank test. miR-139-5p targets vs. others, *p* = 0.036. **B** Comparisons of log_2_-transformed gene expression fold changes of predicted co-target genes of miR-139-5p and other miRNA. Asterisks indicate statistically significant de-repression of target genes as compared to the no-target site. The *p*-value was calculated using the Wilcoxon rank test. miR-139-5p (139) targets vs. no target site, *p* = 0.018. 139 & miR-9 targets, *p* = 0.020. 139 & miR-18 targets, *p* = 0.024. 139 & miR-137 targets, *p* = 0.018. 139 & miR-363 targets, *p* = 0.020. **C** Cumulative distribution plot of log_2_-transformed gene expression fold changes of predicted miR-139-5p target genes and other genes with no target site following miR-139-5p mimics in Lu134A cells. Genes with TPM > 1 in the negative control group were analyzed. The *p*-value was calculated using Wilcoxon rank test. miR-139 targets vs. others, *p* = 1.32 × 10^−11^. **D** Upper: Venn diagram showing an overlap between the downregulated genes by miR-139-5p mimics and predicted target genes of any of four NEUROD1-associated miRNAs (miR-9, miR-18, miR-137, and miR-363). Lower: Venn diagram showing overlap between co-target genes of miR-139-5p and the four NEUROD1-associated miRNAs and ASCL1 target genes confirmed using CUT&Tag for ASCL1 in Lu134A cells. Numbers of genes are indicated. **E** Schematic representation of NEUROD1-, ASCL1-, and miRNA-mediated gene regulation. NEUROD1 (blue) regulates miR-139-5p (blue) and other NEUROD1-associated miRNAs, which are predicted to target several pathologically relevant genes (purple). ASCL1 (red) regulates additional genes (red), some of which are also targeted by NEUROD1-associated miRNAs.

Finally, we explored the target genes of miR-139-5p and their association with the molecular features of SCLC. First, we evaluated whether *NEUROD1* knockout could lead to de-repression of the predicted miR-139-5p target genes. RNA-seq analysis revealed that the predicted miR-139-5p targets tended to increase after *NEUROD1* knockout (miR-139-5p targets vs. others, *p* = 0.036) (Fig. 6A). *NEUROD1* knockout also led to *PDE2A* downregulation (Fig. 3C), it was postulated that miR-139-5p downregulation due to *NEUROD1* knockout resulted in the de-repression of its target genes.

Previous studies showed that multiple miRNAs cooperatively repress their common target genes [21, 34]. We then examined the expression changes of the common target genes of miR-139-5p and other NEUROD1-associated miRNAs after *NEUROD1* knockout (Fig. 6B). miR-139-5p targets shared with miR-9, miR-18, miR-137, or miR-363 showed significant upregulation compared to non-target genes following *NEUROD1* knockout (miR-139-5p targets vs. no target site, *p* = 0.018; miR-139-5p & miR-9 targets, *p* = 0.020; miR-139-5p & miR-18 targets, *p* = 0.024; miR-139-5p & miR-137 targets: *p* = 0.018; miR-139-5p & miR-363 targets, *p* = 0.020). These findings suggested that miR-139-5p and several other NEUROD1-associated miRNAs actively cooperatively regulate gene regulation.

To further validate these miR-139-5p targets, Lu134A cells were transfected with miRNA mimics, and RNA-seq analysis was performed. Compared to negative control, miR-139-5p mimics cause significant repression of the predicted target genes in Lu134A cells (miR-139-5p targets vs. others, *p* = 1.32 × 10^−11^) (Fig. 6C). We identified 252 mRNAs predicted to be miR-139-5p targets and downregulated by miR-139-5p mimics (Table S14). Of these, 134 genes were also predicted targets of miR-9, miR-18, miR-137, or miR-363, supporting the hypothesis of combinatorial regulation (Fig. 6D). These common targets included pathologically relevant genes, including nuclear factor IA (*NFIA*), *NFIB*, *FOXP1*, and notch receptor 1 (*NOTCH1*) [19, 35–37]. These also included ASCL1 target genes confirmed using CUT&Tag assay in Lu134A cells (Fig. 2C), such as ELAV-like RNA binding protein 2 (*ELAVL2*) and neuronal pentraxin 1 (*NPTX1*) **(**Fig. 6D**)**.

Together, these results indicate that miR-139-5p, along with other NEUROD1-associated miRNAs cooperatively regulate gene expression, potentially contributing to the molecular heterogeneity of SCLC (Fig. 6E).

## Discussion

Recent studies demonstrated SCLC subtype conversion from SCLC-A to SCLC-N by cell culture and genetically engineered mouse models [12–15]. Moreover, it was previously shown that ASCL1 and NEUROD1 were co-expressed in 30 to 40% of SCLC cases [16–18], implying SCLC plasticity. In the current study, we observed immunoreactivity for both ASCL1 and NEUROD1 in almost half of the SCLC tissue samples, which motivated us to characterise ASCL1/NEUROD1 double-positive SCLC. Genome-wide profiling of histone modifications, ASCL1 and NEUROD1 occupancy, gene and miRNA expression, and gene co-expression patterns determined using the CUT&Tag assay, RNA-seq, and small RNA-seq analyses revealed that ASCL1 and NEUROD1 regulate distinct target genes and miRNAs in SCLC-A/N cells.

In addition to the findings of our previous study [21], ASCL1, and NEUROD1 appeared to control SE-associated transcriptional programs, possibly in cooperation with several key transcription factors characteristic of each transcription factor (Supplementary Fig. S4). Our integrative analyses suggested that NEUROD and NHLH transcription factors might constitute unique transcriptional networks in NEUROD1-positive SCLC, which warrants further investigations.

Although the exact mechanism underlying the development of SCLC-A/N remains unclear, SCLC-A/N might represent an intermediate state in the transition process from SCLC-A to SCLC-N. In line with the previous studies showing that MYC facilitates the development of NEUROD1-positive SCLC [12, 13], MYC expression levels in SCLC-A/N (CORL279, H2066, and DMS53) were higher than those in SCLC-A (Fig. 3E).

Taking advantage of ASCL1/NEUROD1 double-positive SCLC cells, we assessed the possibility of reciprocal regulations between ASCL1 and NEUROD1; however, we did not observe statistically significant changes in ASCL1 target genes by *NEUROD1* knockout or in NEUROD1 target genes via *ASCL1* silencing (data not shown) [21]. Our findings indicated that transcriptional programs previously characterised in SCLC-A and SCLC-N subtypes [10] were similarly activated in parallel SCLC-A/N cells.

We expanded the scope of analyses to explore NEUROD1-regulated genes and miRNAs. This is the first study to describe that miR-139-5p and its host gene, *PDE2A*, are tightly associated with NEUROD1; no previous studies have reported an association between SCLC and PDE2A. However, regarding the functional importance of *PDE2A* as suggested by embryonic lethality of *PDE2A* knockout mice [38], PDE2A or miR-139-5p may play functional roles in the pathogenesis of NEUROD1-positive SCLC, as reported in non-small-cell lung cancer cells [39].

Our study is the first to identify candidate target genes of miR-139-5p in SCLC, which included *NFIB* and *NOTCH1*, in cooperation with several other miRNAs (miR-9, miR-18, miR-137, and miR-363). NFIB is a potent driver of SCLC metastasis and is co-expressed with ASCL1 and TTF-1 in SCLC [19, 37, 40]. Given that miR-139-5p might suppress *NFIB* in NEUROD1-positive SCLC, such a mechanism might underlie the phenotypic differences between SCLC-A and SCLC-N. Inactivating *NOTCH1* mutations have been frequently observed in human SCLC tissue samples, and previous studies have highlighted the tumour-suppressive roles of NOTCH1 by cell culture and mouse models [32, 41]. Moreover, NOTCH signalling regulates neuroendocrine differentiation in SCLC [32]. Thus, miR-139-5p might modulate cell features of NEUROD1-positive SCLC by *NOTCH1* suppression.

This study has several limitations. First, the SCLC-A/N subtype was defined solely based RNA expression levels of ASCL1 and NEUROD1, although transcription factor differences were still detectable in publicly available datasets. While subtype-based therapies, such as tarlatamab, which targets DLL3, a downstream target of ASCL1, are moving towards clinical application [42], the concurrent transcriptional activity of ASCL1 and NEUROD1 in SCLC-A/N, as demonstrated in our study, may complicate treatment strategies. This highlights the increasing importance of accurately defining this subtype. Furthermore, we did not directly assess miR-139-5p expression levels in clinical samples; instead, we evaluated the expression of its highly correlated host gene, *PDE2A*, as a surrogate. Future studies should quantitatively assess miR-139-5p expression alongside SCLC subtype to provide a more comprehensive understanding.

In conclusion, this study characterised the molecular features of ASCL1/NEUROD1 double-positive SCLC and demonstrated transcriptional programs differentially regulated by ASCL1 and NEUROD1. Comprehensive profiling of NEUROD1-regulated genes and miRNAs enabled us to illustrate miRNA-mediated combinatorial gene regulation that shapes the phenotype of NEUROD-positive SCLC. Our findings thus shed light on the novel aspect of the gene regulatory network underlying molecular heterogeneity of SCLC.

## Supplementary information

Supplemetary Figures

Supplementary legends

Supplementary tables

## Data Availability

The datasets generated during and/or analysed during the current study are available in the DNA Data Bank of Japan (DRA012871) and the Gene Expression Omnibus repository (GSE270263 and GSE277353).
