# Supplementary material for: Integrative epigenome and transcriptome analyses reveal transcriptional programs differentially regulated by ASCL1 and NEUROD1 in small cell lung cancer
Source: Oncogene. 2025 Jul 1;44(34):3113–25. doi: 10.1038/s41388-025-03481-2 (PMC12358297; doi:10.1038/s41388-025-03481-2)
Supplement: Supplementary file 1 — Supplemetary Figures [file 41388_2025_3481_MOESM1_ESM.pdf]

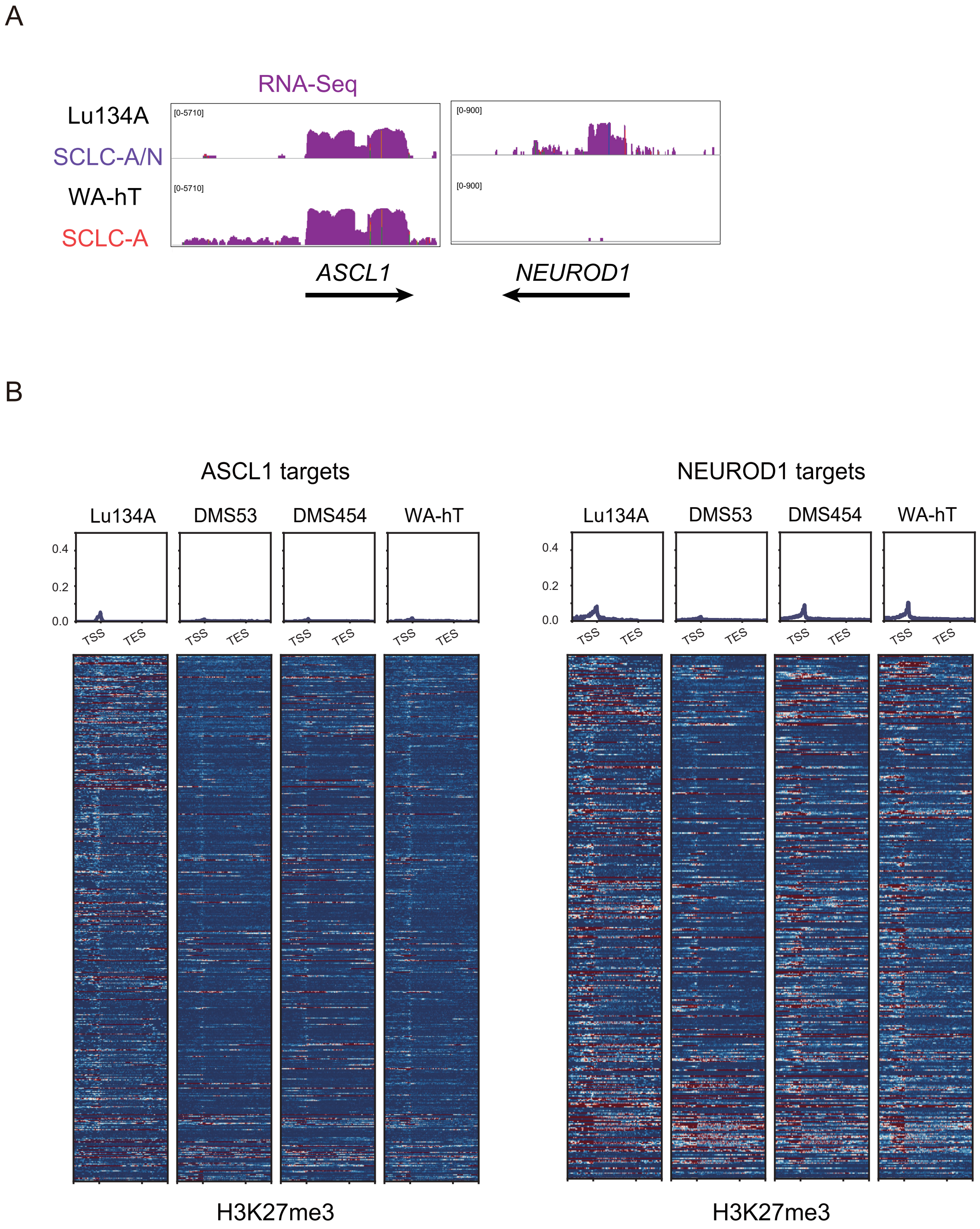
A
RNA-Seq
Lu134A
SCLC-A/N
WA-hT
SCLC-A
[0-5710]
[0-5710]
[0-900]
[0-900]
ASCL1
NEUROD1
B
ASCL1 targets
NEUROD1 targets
Lu134A
DMS53
DMS454
WA-hT
0.4
0.2
0.0
TSS
TES
H3K27me3

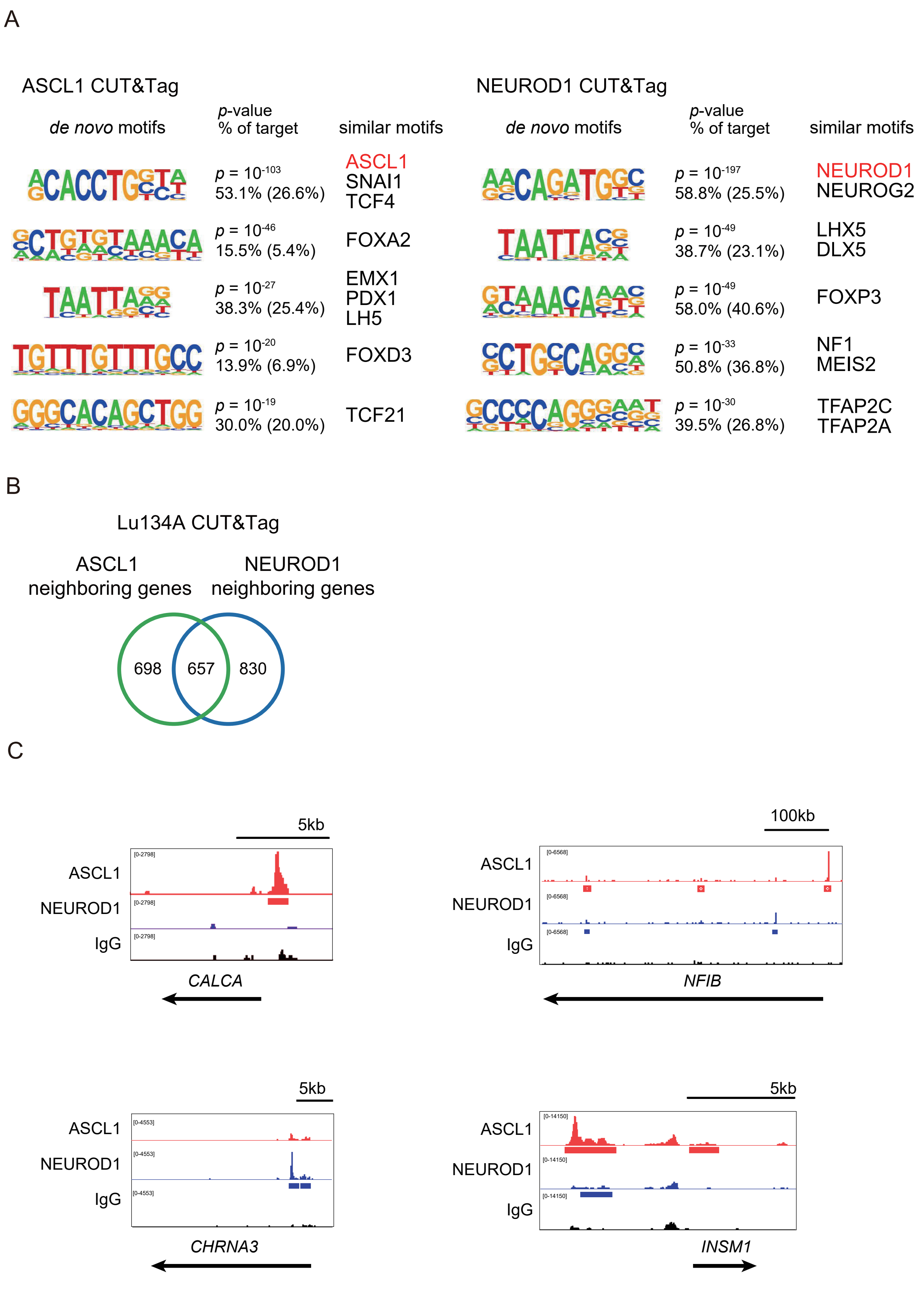
A
ASCL1 CUT&Tag
de novo motifs
p-value
% of target
similar motifs
p = 10-103
53.1% (26.6%)
ASCL1
SNAI1
TCF4
p = 10-46
15.5% (5.4%)
FOXA2
p = 10-27
38.3% (25.4%)
EMX1
PDX1
LH5
p = 10-20
13.9% (6.9%)
FOXD3
p = 10-19
30.0% (20.0%)
TCF21
NEUROD1 CUT&Tag
de novo motifs
p-value
% of target
similar motifs
p = 10-197
58.8% (25.5%)
NEUROD1
NEUROG2
p = 10-49
38.7% (23.1%)
LHX5
DLX5
p = 10-49
58.0% (40.6%)
FOXP3
p = 10-33
50.8% (36.8%)
NF1
MEIS2
p = 10-30
39.5% (26.8%)
TFAP2C
TFAP2A
B
Lu134A CUT&Tag
ASCL1
neighboring genes
NEUROD1
neighboring genes
698
657
830
C
5kb
ASCL1
NEUROD1
IgG
[0-2798]
CALCA
100kb
[0-6568]
NFIB
5kb
[0-4553]
CHRNA3
5kb
[0-14150]
INSM1

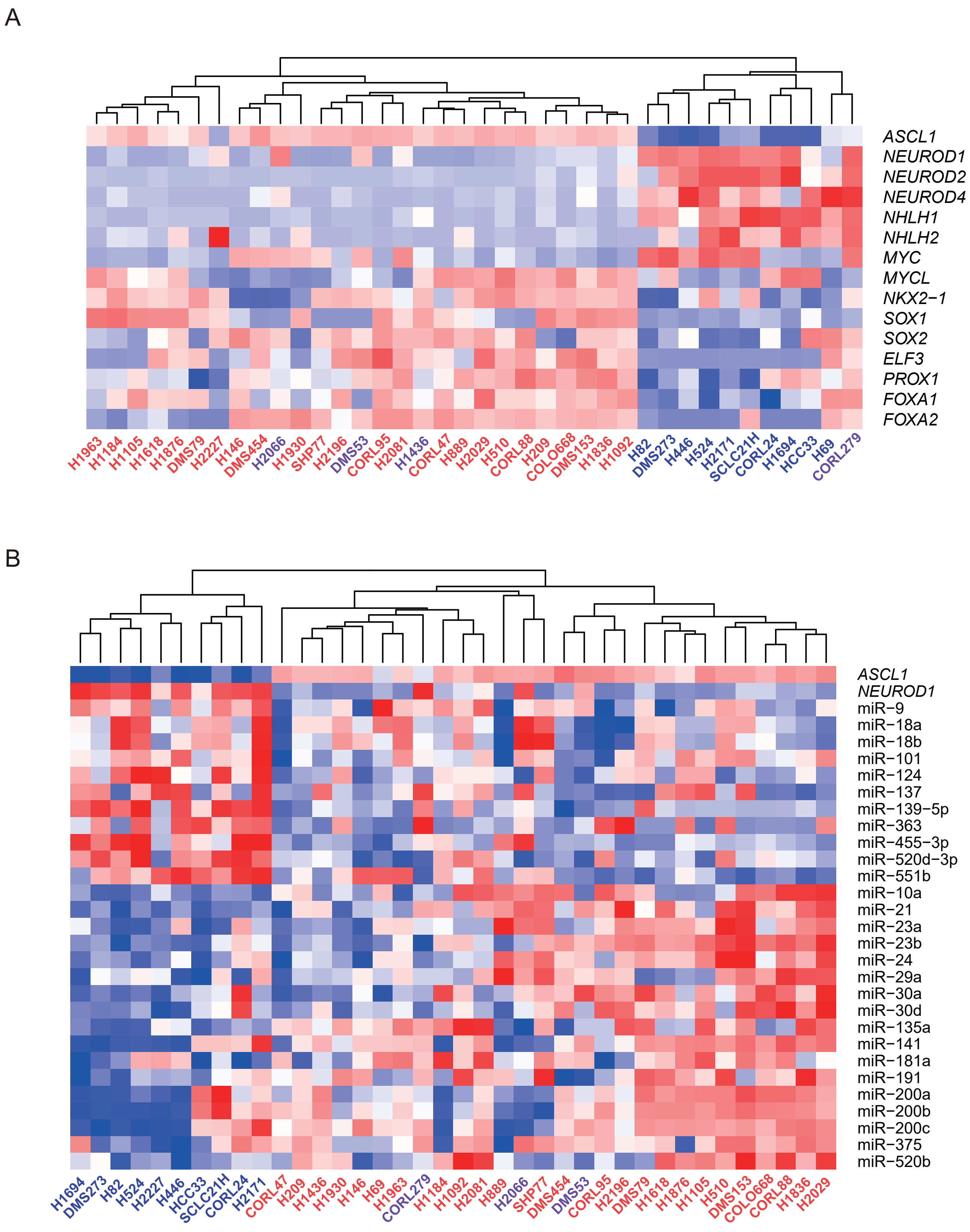
A
ASCL1
NEUROD1
NEUROD2
NEUROD4
NHLH1
NHLH2
MYC
MYCL
NKX2−1
SOX1
SOX2
ELF3
PROX1
FOXA1
FOXA2
H1963
H1184
H1105
H1618
H1876
DMS79
H2227
H146
DMS454
H2066
H1930
SHP77
H2196
DMS53
CORL95
H2081
H1436
CORL47
H889
H2029
H510
CORL88
H209
COLO668
DMS153
H1836
H1092
H82
DMS273
H446
H524
H2171
SCLC21H
CORL24
H1694
HCC33
H69
CORL279
B
ASCL1
NEUROD1
miR−9
miR−18a
miR−18b
miR−101
miR−124
miR−137
miR−139−5p
miR−363
miR−455−3p
miR−520d−3p
miR−551b
miR−10a
miR−21
miR−23a
miR−23b
miR−24
miR−29a
miR−30a
miR−30d
miR−135a
miR−141
miR−181a
miR−191
miR−200a
miR−200b
miR−200c
miR−375
miR−520b
H1694
DMS273
H82
H524
H2227
H446
HCC33
SCLC21H
CORL24
H2171
CORL47
H209
H1436
H1930
H146
H69
H1963
CORL279
H1184
H1092
H2081
H889
H2066
SHP77
DMS454
DMS53
CORL95
H2196
DMS79
H1618
H1876
H1105
H510
DMS153
COLO668
CORL88
H1836
H2029

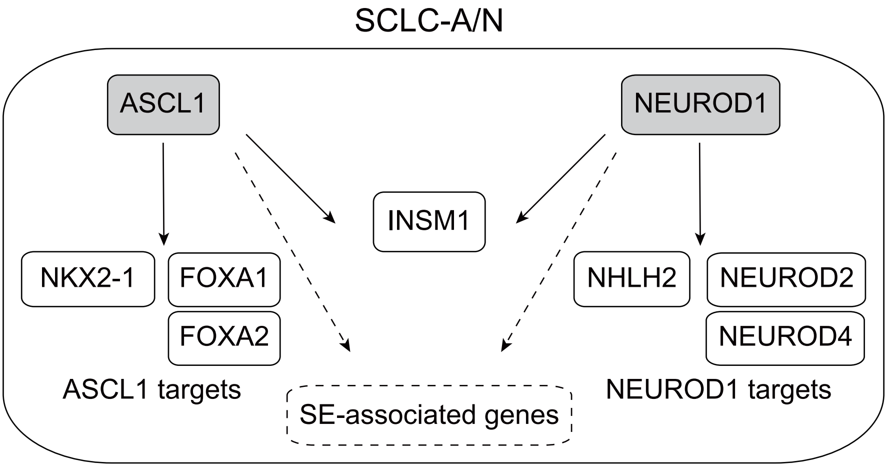
SCLC-A/N
ASCL1
NEUROD1
INSM1
NKX2-1
FOXA1
FOXA2
NHLH2
NEUROD2
NEUROD4
ASCL1 targets
SE-associated genes
NEUROD1 targets
