# Supplementary material for: Integrative epigenome and transcriptome analyses reveal transcriptional programs differentially regulated by ASCL1 and NEUROD1 in small cell lung cancer
Source: Oncogene. 2025 Jul 1;44(34):3113–25. doi: 10.1038/s41388-025-03481-2 (PMC12358297; doi:10.1038/s41388-025-03481-2)
Supplement: Supplementary file 2 — Supplementary legends [file 41388_2025_3481_MOESM2_ESM.docx]

**Supplementary Figures**

**Figure S1. Trends of ASCL1 and NEUROD1 in SCLC cell lines**

A. RNA-seq signals in the genomic regions around the *ASCL1* and *NEUROD1* gene loci in Lu134A and WA-hT cells.

B. Distributions of H3K27me3 signals within the intervals of 3.0 kb upstream of transcription start sites (TSSs), gene bodies, and 3.0 kb downstream of transcription end sites (TESs) of the ASCL1 or NEUROD1 target genes in the DMS53, DMS454, Lu134A, and WA-hT cells. Values on the *Y*-axis of the upper panels indicate normalized read counts.

**Figure S2. ASCL1 & NEUROD1 CUT&Tag analysis in Lu134A cells**

A. Motif analysis for the binding sites of ASCL1 and NEUROD1 identified using the CUT&Tag assay in Lu134A cells. The top five motifs are shown in descending order based on the *p*-value. The frequency of each motif within the target sequences and the background sequence (in parentheses) is given. Similar known motifs are shown on the right.

B. Venn diagram showing neighboring genes of CUT&Tag peaks for ASCL1 and NEUROD1 in Lu134A cells. Numbers of genes are indicated.

C. ASCL1 and NEUROD1 CUT&Tag signals in Lu134A in the genomic regions around the representative gene loci. IgG signals represent the background. The lines represent CUT&Tag signal peaks.

**Figure S3.** **Heatmap of SCLC-A, SCLC-A/N, and SCLC-N transcription factor and miRNA expression in 38 SCLC cell lines.**

A. Heatmap illustrating the relative expression levels of key transcription factors in 38 SCLC cell lines, with hierarchical clustering. The colour of the cell line labels corresponds to the subtype: red for SCLC-A, purple for SCLC-A/N, and blue for SCLC-N.

B. Heatmap showing the relative expression levels of ASCL1, NEUROD1, NEUROD1-associated miRNAs, and ASCL1-associated miRNAs in 38 SCLC cell lines, with hierarchical clustering. The colour of the cell line labels corresponds to the subtype: red for SCLC-A, purple for SCLC-A/N, and blue for SCLC-N.

**Figure S4.** **Schematic diagram illustrating the relationship between ASCL1, NEUROD1, and their respective and shared target genes in SCLC-A/N**

ASCL1 and NEUROD1 are shown alongside their specific downstream targets (e.g. NKX2-1 and FOXA1 for ASCL1; NHLH2 and NEUROD2 for NEUROD1), as well as shared targets such as INSM1 and SE-associated genes.

**Supplementary Tables**

**Table S1.** Public datasets analyzed in this study

**Table S2.** CCLE cell lines analyzed in this study

**Table S3.** Sequences of sgRNA for *NEUROD1* knockout and negative control

**Table S4**. Antibodies used in this study

**Table S5.** Characteristics of clinical samples analyzed for immunohistochemistry

**Table S6.** Human transcription factors

**Table S7.** Target genes of ASCL1 and NEUROD1

**Table S8.** Neighboring genes of the peaks detected using CUT&Tag for ASCL1 or NEUROD1 in Lu134A cells

**Table S9**: Downregulated genes by ASCL1 knockdown in Lu134A cells

**Table S10**: Upregulated and downregulated genes by *NEUROD1* knockout in Lu134A cells

**Table S11:** Genes associated with super-enhancers or typical enhancers defined by H3K27ac CUT&Tag in Lu134A cells

**Table S12**: SE- and TE-associated transcription factors in SCLC cell lines

**Table S13**: Upregulated and downregulated miRNAs by *NEUROD1* knockout in Lu134A cells

**Table S14:** Predicted target genes of miR-139-5p and other miRNAs downregulated by miR-139-5p mimics in Lu134A cells
